# Rootstocks Overexpressing *StNPR1* and *StDREB1* Improve Osmotic Stress Tolerance of Wild-Type Scion in Transgrafted Tobacco Plants

**DOI:** 10.3390/ijms22168398

**Published:** 2021-08-05

**Authors:** Yasmine S. Hezema, Mukund R. Shukla, Alok Goel, Murali M. Ayyanath, Sherif M. Sherif, Praveen K. Saxena

**Affiliations:** 1 Gosling Research Institute for Plant Preservation, Department of Plant Agriculture, University of Guelph, Guelph, ON N1G 2W1, Canada; yhezema@uoguelph.ca (Y.S.H.); mshukla@uoguelph.ca (M.R.S.); alok@uoguelph.ca (A.G.); ayyanath@uoguelph.ca (M.M.A.); 2 Department of Horticulture, Damanhour University, Damanhour 22713, El-Beheira, Egypt; 3 Alson H. Smith Jr. Agricultural Research and Extension Center, School of Plant and Environmental Sciences, Virginia Tech, Winchester, VA 22602, USA

**Keywords:** transgrafting, mRNA transport, osmotic stress, ABA, *StNPR1*, *StDREB1*, ORGs, ROS

## Abstract

In grafted plants, the movement of long-distance signals from rootstocks can modulate the development and function of the scion. To understand the mechanisms by which tolerant rootstocks improve scion responses to osmotic stress (OS) conditions, mRNA transport of osmotic responsive genes (ORGs) was evaluated in a tomato/potato heterograft system. In this system, *Solanum tuberosum* was used as a rootstock and *Solanum lycopersicum* as a scion. We detected changes in the gene expression levels of 13 out of the 21 ORGs tested in the osmotically stressed plants; of these, only *NPR1* transcripts were transported across the graft union under both normal and OS conditions. Importantly, OS increased the abundance of *StNPR1* transcripts in the tomato scion. To examine mRNA mobility in transgrafted plants, *StNPR1* and *StDREB1* genes representing the mobile and non-mobile transcripts, respectively, were overexpressed in tobacco (*Nicotiana tabacum*). The evaluation of transgenic tobacco plants indicated that overexpression of these genes enhanced the growth and improved the physiological status of transgenic plants growing under OS conditions induced by NaCl, mannitol and polyethylene glycol (PEG). We also found that transgenic tobacco rootstocks increased the OS tolerance of the WT-scion. Indeed, WT scions on transgenic rootstocks had higher ORGs transcript levels than their counterparts on non-transgenic rootstocks. However, neither *StNPR1* nor *StDREB1* transcripts were transported from the transgenic rootstock to the wild-type (WT) tobacco scion, suggesting that other long-distance signals downstream these transgenes could have moved across the graft union leading to OS tolerance. Overall, our results signify the importance of *StNPR1* and *StDREB1* as two anticipated candidates for the development of stress-resilient crops through transgrafting technology.

## 1. Introduction

Vascular tissues (i.e., phloem and xylem) extend throughout the whole plant, forming a continuous trafficking pathway that allows for long-distance communication. As part of this communication process, phloem and xylem can transport small molecules such as water, hormone, ions, amino acids, and photoassimilates [1,2,3]. In fact, large molecules like RNA and proteins can move not only intracellularly, but also over long distances, and some of these molecules can work as systemic signals in plants [4,5,6,7,8,9,10]. For example, the mobile florigen protein encoded by the *flowering locus t* (*FT*) gene, which is a part of the signal that is involved in promoting flowering, is produced in the rootstock leaves and moves through the graft union to the growing points of the scion to promote flowering [6]. Besides, different types of RNA (mRNAs, miRNAs, and siRNAs) can be transported over long distances through the graft union, and these mobile RNAs can modulate several functions in the distant target tissue [5,9,11,12,13]. For instance, BEL1-like transcription factor (*BEL5*) mRNA can be transported over a long distance from the scion leaves to the rootstock to regulate tuber formation in potatoes by modulating genes that control growth and activating the sugar transport system to enhance assimilates influx and sink strength of young tubers [4,14,15,16].

Osmotic stress (OS) is a major threat to sustainable crop production worldwide. Almost all abiotic stresses such as drought, salinity, heat, cold, and freezing induce OS [17,18,19,20,21]. OS is characterized by decreased turgor pressure and increased water loss, which causes severe damage to cell membranes, disrupts normal cellular activities, and finally results in plant death. As a tolerance mechanism, plants have evolved different strategies to avoid and mitigate OS [22,23,24,25,26]. At the molecular level, plants can increase their tolerance to OS by manipulating the expression of osmotic responsive genes (ORGs) [24,27,28]. These genes are involved in different physiological and biochemical processes such as plant hormone synthesis and signaling, cell expansion, lateral root formation, and stomatal closure [29,30,31,32,33]. An example of biochemical changes associated with abiotic stress is the induction of abscisic acid (ABA), a known stress hormone that serves several roles in plant growth and development [34]. One of the most important roles of ABA under abiotic stress is controlling stomatal closure to mitigate transpiration and water loss [35,36]. ABA also alleviates stress injury by promoting various stress-related genes that are involved in enhancing plant tolerance to stress [20]. Interestingly, ABA also acts as a long-distance signal that moves from the root to the shoot and vice versa through xylem and phloem [37,38,39].

Grafting has been used as a commercial plant propagation by merging two parts of different plants (the upper part is termed ‘scion’ and the lower part is termed ‘rootstock’). Rootstocks can improve the tolerance of grafted plants to biotic stressors such as fire blight, fusarium wilt, phytophthora blight and root-knot nematodes in different plant species [40,41]. Rootstocks are also used to improve plant’s tolerance to abiotic stresses, such heat, cold, salinity and drought stresses [41,42,43], and control the growth and yield of fruit crops [44]. Transgrafting is defined as grafting a wild-type scion to a genetically modified rootstock (or vice versa) to modulate certain aspects of the scion, including tolerance to biotic and abiotic stress [45]. At the commercial level, transgrafting is perceived as a more consumer-friendly technology for germplasm development compared with genetic transformation In essence, transgrafting can improve the qualitative and quantitative agricultural traits of the scion without integrating novel genetic materials in the scion tissues [3,46]. From a scientific perspective, transgrafting provides a useful tool for understanding how large molecules move over long distances and influence distant recipient tissues. In fact, transgrafting has been used to study the underlying mechanisms of the signals involved in dwarfing of tobacco, tomato, and apple plants [47,48,49,50]. In a study using a wild-tobacco scion grafted to a transgenic rootstock expressing the *gai* gene, *gai* transcripts were found in the scion. The translated product of the *gai* mRNA was also found in the scion of the semi-dwarf phenotype, which provides an explanation, at least partially, of how the dwarfing rootstock can decrease the size of the scion [49]. In a similar example, a non-transgenic apple scion grafted to a transgenic apple rootstock expressing *rolB* (rooting locus B) has shown reduced growth and flowering with no effects on fruit quality [44]. The aforementioned study also found that *rolB* transcripts were not detected in non-transgenic apple scion grafted on transgenic apple rootstock. In addition, *Prunus persica* C-repeat binding factor (*PpCBF*) involved in cold and drought stress responses reduced scion growth and delayed flowering of the ‘Royal Gala’ wild-type scion when overexpressed in M26 apple rootstock. However, *PpCBF1* mRNA was not detected in the scion tissues [46].

Genetically modified rootstocks have proven to be efficient for modulating wild-type scion growth [46,49,50]; however, it remains largely unknown how rootstocks can modulate scions’ responses, especially under OS. To the best of our knowledge, there are no previous studies that examined the transport of ORG transcripts over long distances in osmotically stressed grafted plants. Identification of highly regulated ORGs that are involved in promoting OS tolerance in plants can be used to develop tolerant transgenic rootstocks. In the present study, we hypothesized that mRNA of some ORGs could move long distances over graft junctions from the rootstock to the scion tissues under OS conditions. We also hypothesized that transgrafted plants overexpressing mobile ORGs in the rootstock could increase the tolerance of WT scions to OS. Therefore, in the present study we aimed to identify ORGs with mobile mRNA, and investigate the physiological, biochemical and molecular mechanisms underlying the imparted tolerance, if any, from transgenic rootstocks to WT scions in transgrafted plants under OS. To this end, a homograft of potato/potato was used to initially identify ORGs genes whose expression was highly regulated under OS conditions. A heterograft composed of tomato as the recipient scion and potato as the donor rootstock was then used as a model system to assess the translocation of ORGs mRNAs from the rootstock to the scion using qRT-PCR. Two genes involved in two different signaling pathways (*StNPR1* and *StDREB1*) were transformed into tobacco plants. Transgrafts composed of tobacco plants overexpressing *StNPR1* and *StDREB1* as rootstocks and wild-type tobacco (WT) as scions were then used to explore the effects of tolerant transgenic rootstocks on physiological responses, ABA content and gene expression in WT scion under normal and OS conditions.

## 2. Results

### 2.1. Expression Profiles of ORGs in the Homografted Potato Plants

**Figure 1:**
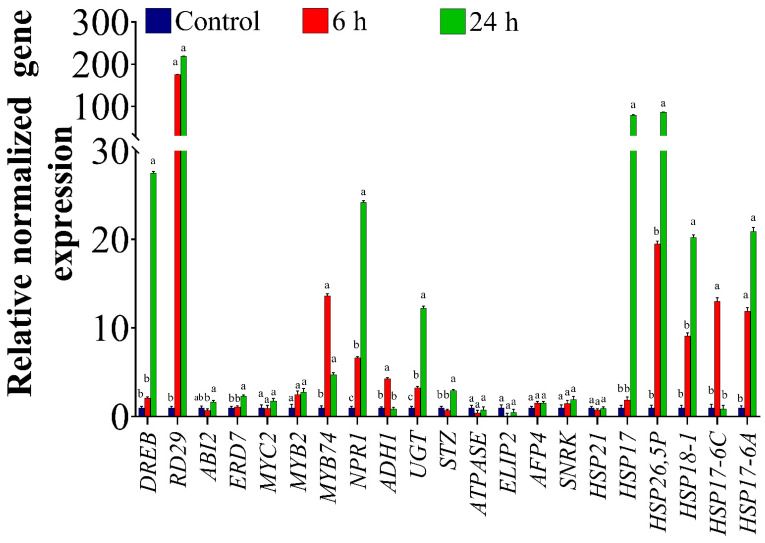
Expression of ORGs genes in homografted potatoes (*Solanum tuberosum*), assessed by qRT-PCR. Plants were osmotically stressed by watering with 30% PEG four weeks after graft establishment. Tissues located 5 cm above the graft union were collected from untreated plants (control) and PEG-treated plants at 6 h and 24 h of treatment. The bars show the normalized relative expression of potato ORGs. The gene expression values were calculated relative to homografted potato control (P/P) and normalized to *TIP*, an internal control. The bars represent the means ± SEM of three biological replicates. Different letters in the graph indicate significant differences (*p* < 0.05) in relative gene expression between treatments after Tukey’s test.

To investigate the expression pattern of ORGs in response to OS in the homografted potato (P/P) plants, a total of 21 highly regulated ORGs [24,32,51] involved in different processes were tested. The results of the qRT-PCR analysis showed differential expression of the selected genes under OS. As shown in Figure 1, compared to the control plants (C-P/P), the level of *dehydration responsive element binding* (*DREB*) transcripts increased by 2- and 27-fold in the scion of the potato 6 h and 24 h, respectively, after PEG treatment. There was also a significant up-regulation (nearly 200-fold) in the expression of the *responsive to desiccation* (*RD29*) gene after 6 h and 24 h compared to the control (Figure 1). The results also showed a significant 2-fold increase in *early responsive to dehydration 7* transcript levels (*ERD7*) at 24 h. On the other hand, transcript levels of the *abscisic acid insensitive 2* (*ABI2*) gene, a negative regulator of abscisic acid (ABA), showed a non-significant reduction at 6 h of PEG treatments, then increased again matching the control at 24 h (Figure 1). There were no changes in the expression levels of *MYC2* and *MYB2.* However, *MYB74* gene expression levels were significantly up-regulated by nearly 13- and 5-fold at 6 h and 24 h, respectively, relative to the control. A 6- and a 24-fold increase in transcript levels was also detected for the *non-expressor of Pathogenesis-Related (PR) genes 1* (*NPR1*) at 6 h and 24 h, respectively (Figure 1). There was also a significant increase in the expression levels of *Alcohol dehydrogenase 1* (*ADH1*) by 4-fold at 6 h (Figure 1). The expression levels of *UDP-sugar glycosyltransferases* (*UGT*) were up-regulated by 3- and 12-fold at 6 h and 24 h, respectively. Additionally, transcript levels of *salt tolerance zinc finger* (*STZ*) increased significantly by 3-fold at 24 h (Figure 1). On the other hand, there were no significant changes in the expression levels of *ATPase*, *LIP2*, *AFP4*, and *SNRK* compared to the control either at 6 h or 24 h (Figure 1). The gene expression levels of six heat shock proteins (*HSP*) were also determined. *HSP17* and *HSP26,5P* showed a similar pattern in their responses, and significantly increased by 80- and 86.6-fold, respectively, at 24 h only. On the other hand, no changes were observed in the abundance of *HSP21* as a result of the stress treatment (Figure 1). *HSP18-1* showed a significant 20-fold increase at 24 h compared to the control (Figure 1). There was also a significant increase of 13-fold in *HSP17-6C* expression at 6 h, after which the levels decreased at 24 h and were similar to the expression levels in the control (Figure 1). *HSP17-6A* demonstrated a significant up-regulation of 12- and 20-fold at 6 h and 24 h, respectively (Figure 1).

### 2.2. The Movement of ORGs Transcripts across the Graft Union under Osmotic Stress Conditions

A strategy based on the grafting of two different plant species was used to identify the genes with mobile transcripts. A heterograft system, in which tomato (*Solanum lycopersicum* ‘Beefsteak’) was used as a scion and potato (*Solanum tuberosum* ‘Gold rush’) as a rootstock, was established to identify the root-to-shoot long-distance movement of mRNAs under normal and osmotic conditions using potato-specific primers. As shown in Supplementary Figure S1, the three potato-specific housekeeping genes (*StGTP, StTIP,* and *StClathrin*) had no detectable expression in scion tissues, indicating their inability to move from the rootstock to scion. Another set of primers (*GTP, TIP,* and *Clathrin*) was designed from conserved nucleotide sequences in tomato and potato to be used for gene expression studies in the heterografts. Of these, only *TIP* showed a gene expression that was stable in potato and tomato under all the tested conditions (Supplementary Figure S1) and was therefore used to normalize the relative gene expression data in both potato and tomato.

mRNA mobility of osmotic-related genes was examined by qRT-PCR using primers that were specific to potato (Supplementary Table S1). The abundance of potato transcripts in the tomato scion examined 6 h after stress induction is shown as a heatmap in Supplementary Figure S2. In general, all ORGs have been detected in the homografted potato (P/P) under control and stress conditions, whereas no transcripts were detected in the homografted tomato (T/T) (Supplementary Figure S2), which confirms the high specificity and reliability of the chosen potato primers. The results also indicated that all, except one of the tested ORGs, have non-mobile transcripts under normal or stress conditions. Only the mRNA of *StNPR1* from the potato rootstock was detected in the tomato scion under normal and OS conditions. The movement through the graft union of different mRNAs encoding the NPR family was then assessed to explore whether the members of the same gene family share the same mRNA mobility properties. The results as presented in Supplementary Figure S2 showed that all the members of the NPR family, except *NPR1* were found to be non-mobile under both normal and stress conditions. This result shows that different members of the same gene family may not follow the same pattern in their transport.

**Figure 2:**
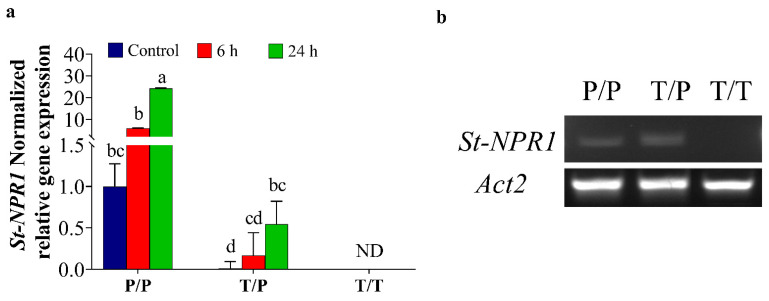
Quantitative and semi-quantitative expression of *StNPR1* in tomato scion. The plants were stressed by watering with 30% PEG. A tissue sampled 5 cm above the graft union at 6 h and 24 h after PEG treatment was used for RNA extractions and gene expression studies. (**a**) The normalized relative gene expression of *StNPR1*. The bars represent the means ± SEM of three biological replicates. The relative expression of potato genes was calculated relative to the homografted potato control (P/P) and normalized to *TIP*. Different letters in the graph indicate significant differences (*p* ≤ 0.05) in relative gene expression between treatments after Tukey’s test, ND means no detected transcripts. (**b**) Semi-quantitative PCR results show the movement of StNPR1 transcripts from the potato rootstock into the tomato scion using potato-specific primers. Homografted potato (P/P), heterografted tomato scion/potato rootstock (T/P), and homografted tomato (T/T). *Actin2* was used as a reference gene.

It is worth noting that although *StNPR1* was detected under both normal and stress conditions, the level of *StNPR1* expression was significantly higher at 24 h in stressed tomato/potato heterografts (T/P) compared to the heterograft control (T/P) (Figure 2a). Compared to the expression of *StNPR1* in the potato homograft control (P/P) (Figure 2a), the *StNPR1* gene expression levels in tomato/potato heterograft (T/P) showed 0.01-fold, 0.17-fold, and 0.55-fold in control, and at 6 h and 24 h, respectively. These results were further confirmed by semi-quantitative RT-PCR, in which primers amplifying a larger *NPR1* fragment (501 bp) were used (Figure 2b).

### 2.3. Potato Rootstock Increased ABA Content in Tomato Scion

**Figure 3:**
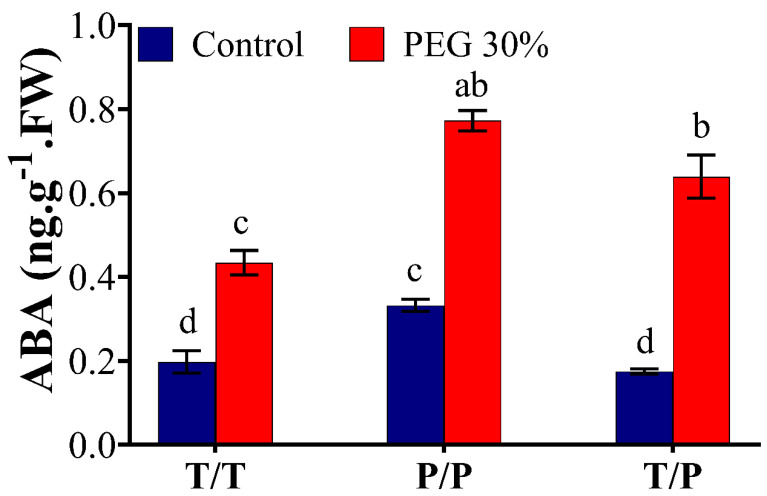
Quantification of ABA in potato and tomato plants. The homografted and heterografted plants were osmotically stressed by watering with 30% PEG. Leaves were collected at 24 h from the scion of homografted tomato (T/T), homografted potato (P/P) and heterografted (T/P) growing under untreated (control) and osmotic stress conditions. The bars represent the means ± SEM of three biological replicates. Different letters in each column indicate significant differences (*p* ≤ 0.05) between treatments after Tukey’s test.

ABA content was determined in the scion leaves of T/T, P/P, T/P plants under control and OS conditions (Figure 3). Within the same treatment, a marked increase of 53%, 57%, and 72% was observed in the leaves of T/T, P/P and T/P, respectively, 24 h after exposure to 30% PEG. ABA level was 33% higher in the leaves of P/P scion relative to T/T under control conditions; however, no significant differences were observed between scions of T/T and T/P plants under control conditions. ABA content also showed 44% and 32.8% increase in the leaves of P/P and T/P, respectively, compared to T/T under OS, suggesting that potato generally has higher ABA content than tomato under normal and stress conditions (Figure 3).

### 2.4. Molecular Analyses of Transgenic Tobacco Plants

**Figure 4:**
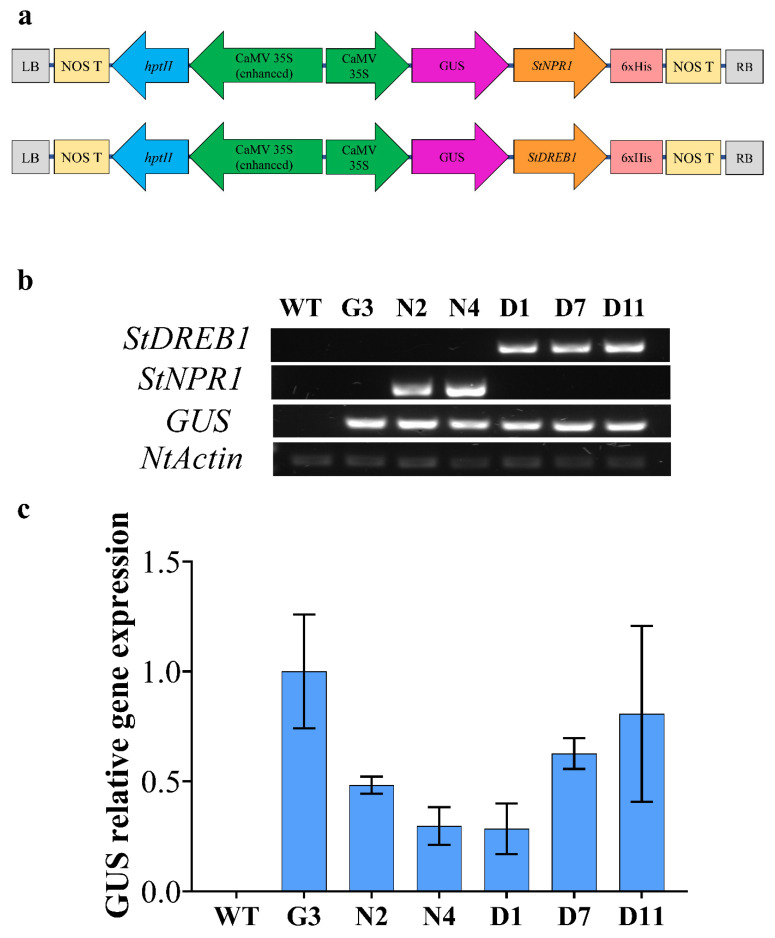
Overexpression of tomato *StNPR1* and *StDREB* genes in tobacco. (**a**) Schematic map of genetic constructs expressing *GUS-StNPR1*- and *GUS-StDREB1* chimeric proteins under the control of the *CaMV35S* promoter. (**b**) Confirmation of transgenes in the genomic DNA by semi-quantitative PCR amplification of *GUS*, *StNPR1* and *StDREB1* transcripts in WT, and transgenic lines of *GUS* only (G3) *StNPR1* (N2 and N4) and *StDREB1* (D1, D7 and D1). Tobacco *Nt-Actin* was used as an internal gene control. (**c**) Relative *GUS* expression in the transgenic lines using qRT-PCR. No significant differences were observed among the transgenic lines at *p* ≤ 0.05 according to Tukey test.

Two pCambia1301plant expression vectors harboring the coding regions of *StNPR1* and *StDREB1* genes under the control of the CaMV35S promoter were constructed, as illustrated in Figure 4a. These vectors were designed to express *GUS* as a reporter gene attached to both transgenes in the same open reading frame (ORF) to facilitate tracking gene transport from transgenic rootstocks to non-transgenic, wild type (WT), scion. Two lines overexpressing *StNPR1* (N2 and N4), three lines overexpressing *StDREB1* (D1, D7 and D11), one line overexpressing *GUS* only (G3) and wild type (WT) tobacco plants were screened by semi-quantitative PCR using *GUS*, *StNPR1* and *StDREB1* primers. Figure 4b confirmed the presence of the transgenes in the genomic DNA of selected F_1_ lines using *NtActin* as an internal reference gene. The relative gene expression analysis using quantitative real-time PCR (qRT-PCR) also revealed high transcript levels of *GUS* in all the transgenic lines G3, N2, D7 and D11, but not in the WT (Figure 4c).

### 2.5. Overexpression of StNPR1 and StDREB1 Confers Enhanced Growth under Stress Conditions

**Figure 5:**
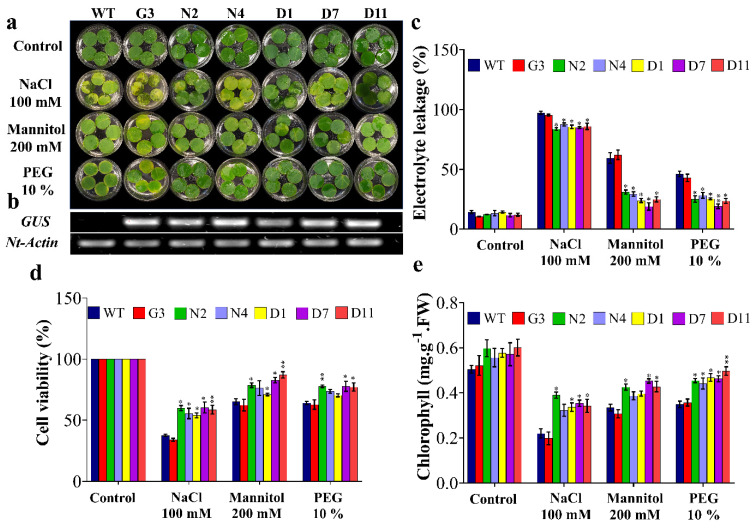
Leaf disc senescence assay and physiological analyses of transgenic lines under stress conditions. (**a**) The leaf discs of F_1_ transgenic lines and WT plants were incubated under NaCl (100 mM), mannitol (200 mM) and PEG (10%) stresses. Control refers to leaf discs at 0 days; stress treatments show leaf discs after nine days of incubation. (**b**) Overexpression of *GUS* in transgenic lines compared to wild-type control plants. (**c**) Estimation of the electrolyte leakage percentage, (**d**) cell viability, (**e**) total chlorophyll. The bars represent mean values ± SEM of three biological replicates (four plants each). Asterisk(s) indicate(s) significant differences between the means of wild type (WT) and transgenic line of each treatment group at * *p* < 0.05, ** *p* < 0.01 according to Dunnett test.

Leaf disc senescence assay was performed to evaluate the tolerance of F_1_ transgenic lines to salinity induced by 100 mM NaCl and OS induced by 200 mM mannitol and 10% PEG for nine days (Figure 5a). In general, the results showed induced damage in leaf discs under various stress treatments; however, the transgenic lines N2, N4, D1, D7 and D11 showed less injuries compared to control plants (WT and G3) under salt and OS conditions. Along the same vein, transgenic lines (N2, N4, D1, D7 and D11) showed an average of 13%, 58% and 43% lower electrolyte leakage (EL) under stress induced by NaCl, mannitol and PEG, respectively, compared to the average of control plants (WT and G3) (Figure 5c). This was also consistent with the cell viability (CV) data, where control plants showed a reduction of 36.4%, 23.2% and 17% in cell viability compared to the transgenic lines exposed to NaCl, mannitol and PEG, respectively (Figure 5d). However, no significant differences were observed in N4 exposed to mannitol and N4 and D1 exposed to PEG (Figure 5d). Another indicator of transgenic plants’ tolerance to OS was revealed by the measurement of leaf’s chlorophyll content. As shown in Figure 5e, transgenic lines, mainly N2, D7 and D11, showed 44.4%, 25.6% and 25.5% higher chlorophyll content under stress induced by NaCl, mannitol and PEG, respectively, compared to WT. Overall, these results illustrated that tobacco plants overexpressing *StNPR1* and *StDREB1* had a better ability to tolerate salt and OS.

**Figure 6:**
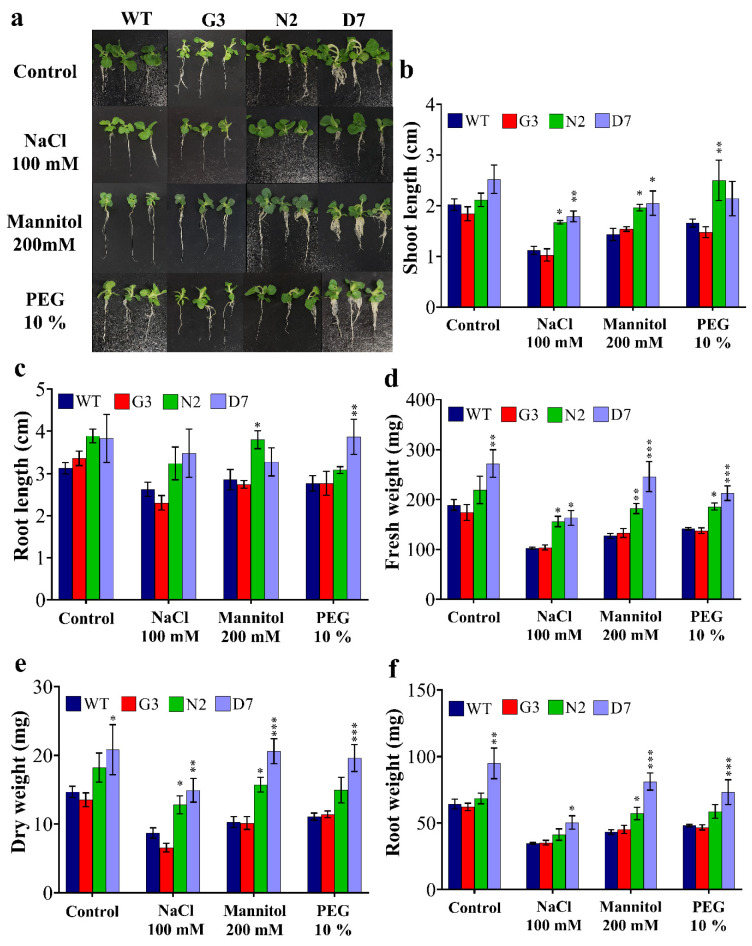
Morphology and growth characteristics of WT and transgenic lines as affected by different stress conditions. (**a**) Fourteen-days seedlings from WT and transgenic lines were grown in vitro for four weeks under control, 100 mM NaCl, 200 mM mannitol and 10% PEG. (**b**) Shoot length, (**c**) root length, (**d**) fresh weight, (**e**) dry weight and (**f**) root fresh weight. The bars represent mean values ± SEM of three biological replicates (four plants each). Asterisk(s) indicate(s) significant differences between the means of wild type (WT) and transgenic line of each treatment group at * *p* < 0.05, ** *p* < 0.01, *** *p* < 0.001 according to Dunnett test.

Based on leaf disc assay results (Figure 5), the F_2_ transgenic lines (G3, N2 and D7) and WT were selected for further morphological and biochemical analyses. The seed germination of G3, N2 and D7 transgenic lines was checked on MS + vitamins medium supplemented with 20 µg/mL hygromycin for two weeks, and the surviving seedlings were selected for further characterization. The surviving seedlings were then sub-cultured for four weeks on MS media supplemented with 100 mM NaCl, 200 mM, 10% PEG and MS + vitamins for WT plants (Figure 6a). Figure 6b shows a significant improvement of shoot length in N2 and D7 under salt and OS compared to control group (WT and G3) grown under the same conditions. Indeed, N2 showed 34.1%, 25% and 40% higher shoot length under NaCl, mannitol and PEG stresses, respectively. Similarly, D7 demonstrated 44.1%, 29.3% and 27.6% higher shoot length under NaCl, mannitol and PEG stresses, respectively, compared to control plants. As far as root growth is concerned, our data showed that root length was significantly higher in N2 and D7 under induced OS by mannitol and PEG stresses, respectively (Figure 6c). Additionally, we found that N2 expressed 34%, 28.6% and 24.9% higher fresh weight under NaCl, mannitol and PEG stresses, respectively, compared to the control plants. Similarly, D7 showed higher fresh weight by 33%, 36.8%, 47.2% and 43.7% under control, NaCl, mannitol and PEG conditions, respectively, compared to WT (Figure 6d). In the same manner, dry weight in N2 seedlings was 40.7%, 35% and 25.3% higher under NaCl, mannitol and PEG conditions, respectively (Figure 6e); while D7 was 48.3%, 50.5% and 42.9% higher under the same conditions, respectively. Moreover, root weight was significantly higher in D7 plants by 33.7%, 30%, 45.8 and 35.2% growing under control, NaCl, mannitol and PEG conditions, respectively, while N2 plants showed 22.8% higher root weight compared to control plants when exposed 200 mM mannitol (Figure 6f). Among salt and OS treatments, N2 and D7 exhibited better growth under OS than under salt stress. The selected transgenic lines were generally healthier and showed better growth parameters under salt and OS than WT and G3.

### 2.6. Transgenic Rootstocks Improved the Growth of WT Scion in Transgrafted Tobacco Plants

**Figure 7:**
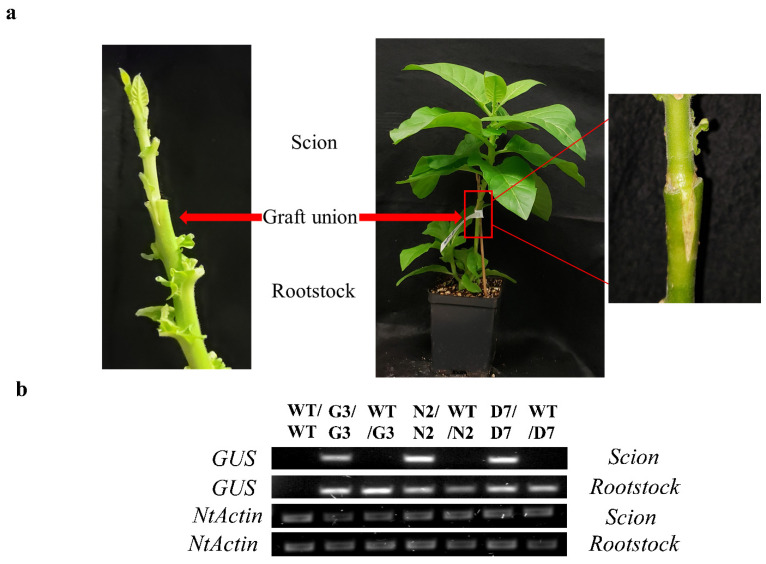
Analysis of transgrafted tobacco plants under osmotic stress conditions. (**a**) Wedge grafting and graft union four weeks from grafting. (**b**) Semi-quantitative PCR amplification of *GUS* in the scion of various graft union; WT/WT, G3/G3, WT/G3, N2/N2, WT/N2, D7/D7 and WT/D7. *NtActin* was used as an internal gene control.

**Figure 8:**
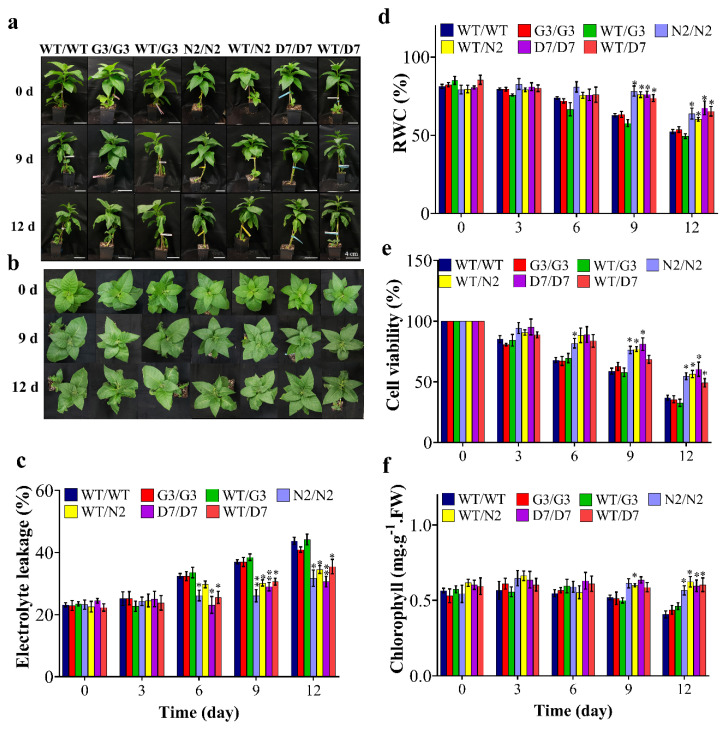
Physiological analysis of transgrafted tobacco plants under osmotic stress conditions. Comparison of wild-type homografted and transgrafted plants at 0, 3, 6, 9 and 12 days of exposing to PEG 10%; WT/WT, G3/G3, WT/G3, N2/N2, WT/N2, D7/D7 and WT/D7 are grafting combinations. (**a**,**b**) Tobacco transgrafted plants response after 0, 9 and 12 days of PEG treatment. (**c**) Electrolyte leakage, (**d**) relative water content (RWC), (**e**) cell viability, (**f**) chlorophyll content of homografts and transgrafts exposed to osmotic stress (10% PEG). The bars represent mean values ± SEM of three biological replicates (four plants each). Asterisk(s) indicate(s) significant differences between the means of wild type (WT/WT) and transgrafts of each treatment group at * *p* < 0.05, ** *p* < 0.01 according to Dunnett test.

**Figure 9:**
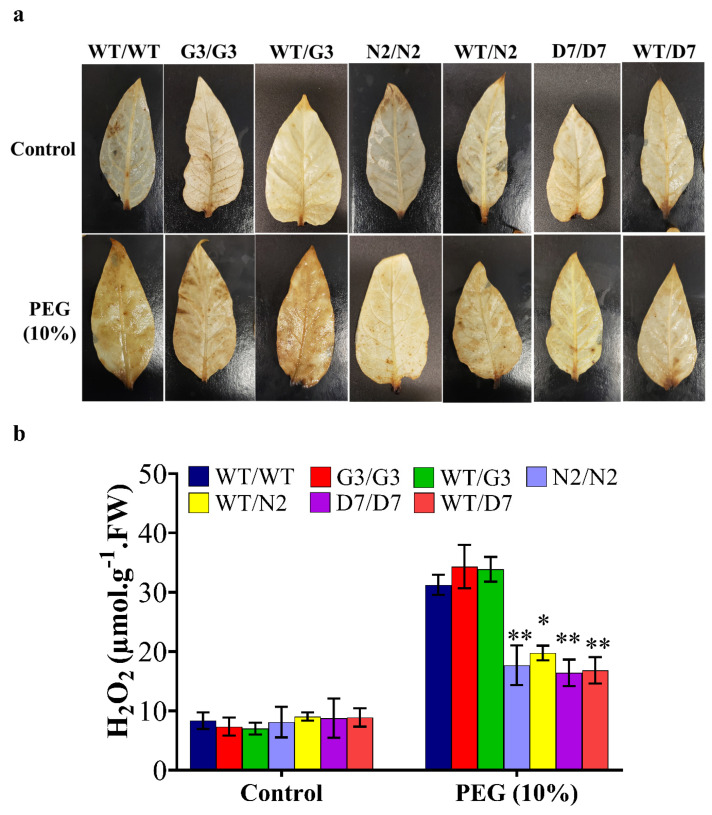
Celluar H_2_O_2_ content in the leaves of WT and transgrafted plants under osmotic stress conditions. Histochemical localization (**a**) and quantification of hydrogen peroxide content (**b**) in leaves of transgrafted tobacco plants; WT/WT, G3/G3, WT/G3, N2/N2, WT/N2, D7/D7 and WT/D7 are grafting combinations. The bars represent mean values ± SEM of three biological replicates (four plants each). Asterisk(s) indicate(s) significant differences between the means of wild type (WT/WT) and transgrafts of each treatment group at * *p* < 0.05, ** *p* < 0.01 according to Dunnett test.

N2 and D7 overexpressing *StNPR1* and *StDREB1*, respectively, were used as rootstocks to study the effect of the tolerant transgenic rootstocks on the WT scions in transgrafted tobacco plants (Figure 7a) exposed to 10% PEG. We initially examined the transport of transcripts of *StNPR1* and *StDREB1* from the corresponding transgenic rootstocks to the non-transgenic WT, scions by semi-quantitative PCR. We found that the transcripts of neither *StNPR1* nor *StDREB1* were detected in WT-scion of WT/N2 or WT/D7 transgrafted treatments (Figure 7b). Figure 8a,b shows the morphological changes in seven groups of grafted plants after exposure to 10% PEG for 0 day (control), 9 and 12 days. These groups represent homo- and heterografts of WT and transgenic plants, expressed as scion/rootstock. For instance, N2/N2 refers to a homograft of transgenic N2 plants, whereas WT/N2 refers to a heterograft of WT scion on N2 rootstock. The significant changes in physiological parameters between transgrafts and WT/WT appeared in samples collected at 9 and 12 days only. All the seven grafting treatments conferred similar responses under control conditions. In general, WT/WT, G3/G3 and WT/G3 plants showed comparable responses under OS conditions as indicated by relative water content (RWC), EL, CV and total chlorophyll content (Figure 8c–f). Similarly, N2/N2, D7/D7, WT/N2, and WT/D7 plants exhibited comparable responses after exposure to 10% PEG (Figure 8c–f). The use of tolerant transgenic rootstocks (N2 and D7) improved physiological parameters of WT scion in transgrafted tobacco (WT/N2 and WT/D7) compared to WT/WT under OS. Membrane injury measured by EL was lower in the scion of N2/N2, D7/D7, WT/N2 and WT/D7 than control plants under stress conditions at 9 and 12 days (Figure 8c). Furthermore, transgrafted plants showed significantly higher RWC and CV than the control group (WT/WT, G3/G3 and WT/G3) at 9 and 12 days after being subjected to OS (Figure 8d,e). Total chlorophyll content was also significantly higher in transgrafted tobacco compared to control plants at 12 days of stress treatment (Figure 8f). The cellular hydrogen peroxides (H_2_O_2_) content indicated by the DAP staining or the titanium chloride method showed more accumulation of H_2_O_2_ in the leaves of the control plants compared to N2/N2, WT/N2, D7/D7 and WT/D7 under OS conditions (Figure 9). The results of EL, RWC, CV, chlorophyll content and H_2_O_2_ content provided evidence that using tolerant transgenic rootstocks overexpressing *StNPR1* and *StDREB1* could improve the growth of WT-scion compared to WT/WT growing under OS conditions.

### 2.7. Evaluation of ABA Content in Transgrafted Tobacco Plants Growing under Osmotic Stress

**Figure 10:**
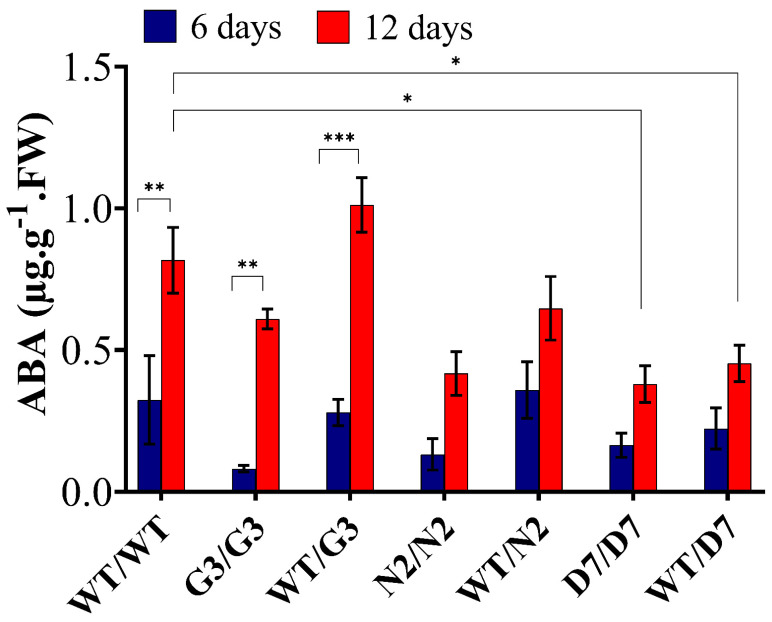
ABA quantification in the leaves of transgrafted tobacco plants under osmotic stress conditions. Abscisic acid (ABA) content was determined in the scion leaves 6 and 12 days of exposure to osmotic stress (10% PEG). WT/WT, G3/G3, WT/G3, N2/N2, WT/N2, D7/D7 and WT/D7 are grafting combinations. The bars represent mean values ± SEM of three biological replicates (four plants each). Asterisk(s) indicate(s) significant differences between the means of wild type (WT/WT) and transgrafts of each treatment group at * *p* < 0.05, ** *p* < 0.01, *** *p* < 0.001 according to Dunnett test.

We determined the amount of abscisic acid (ABA) at 0, 6 and 12 days of OS treatment (Figure 10) to evaluate whether ABA level in the scion could be changed in relation to the OS as well as the rootstock’s level of ABA. In general, ABA content was not detectable at zero-day. However, at 12 days of exposure to 10% PEG, ABA level significantly increased in WT/WT, G3/G3 and WT/G3 by 68%, 83% and 73%, respectively, compared to its level at six days. In addition, N2/N2, WT/N2, D7/D7 and WT/D7 showed an increase of 65%, 45%, 58% and 53%, respectively, but such increase was not statistically significant. When compared to WT/WT, ABA level at 12 days in N2/N2, WT/N2, D7/D7 and WT/D7 were lower by 48.7%, 28%, 53.7% and 42.6%, respectively.

### 2.8. Evaluation of Osmotic-Responsive Gene Expression in Transgrafted Tobacco Plants Grown under Osmotic Stress

**Figure 11:**
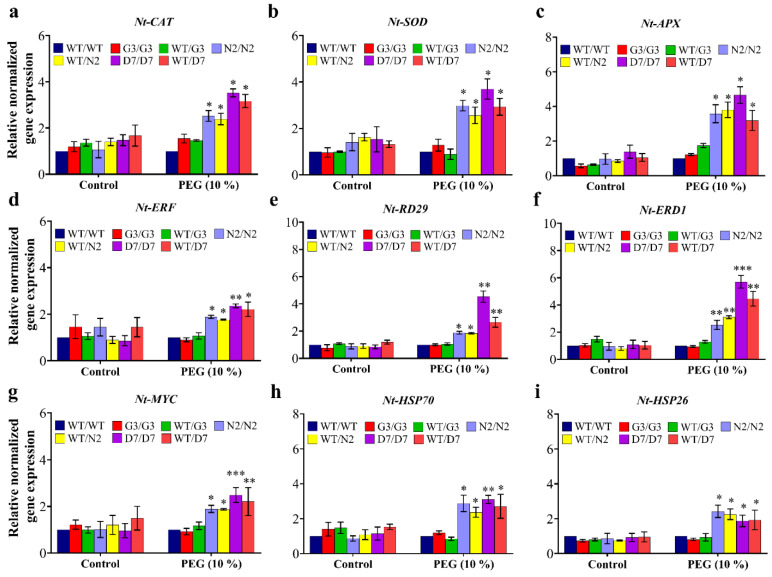
Gene expression analysis in transgrafted tobacco plants under osmotic stress conditions. Normalized relative gene expression of (**a**) *NtCAT*, (**b**) *NTSOD*, (**c**) *NtAPX*, (**d**) *NtERF*, (**e**) *NtRD29*, (**f**) *NtERD1*, (**g**) *NtMYC*, (**h**) *NtHSP70*, and (**i**) *NtHSP26* genes three days after stress treatment. Genes differentially expressed in plants under stress conditions were normalized with *NtEF1*. WT/WT, G3/G3, WT/G3, N2/N2, WT/N2, D7/D7 and WT/D7 are grafting combinations. The bars represent mean values ± SEM of three biological replicates (four plants each). Asterisk(s) indicate(s) significant differences between the means of WT/WT and transgrafts of each treatment group at * *p* < 0.05, ** *p* < 0.01, *** *p* < 0.001 according to Dunnett test.

The gene expression of three reactive oxygen species (ROS) detoxification genes (*NtCAT*, *NtSOD* and *NtAPX*) and six osmotic response genes *(NtERF*, *NtRD29*, *NtERD1*, *NtMYC*, *NtHSP70* and *NtHSP26*) was studied in transgenic and wild-type tobacco plants after three days of PEG-induced stress. In general, the gene expression of all the tested genes was statistically similar among all tobacco gentotypes under control (no-stress) conditions. In addition, G3/G3 and WT/G3 and WT/WT plants showed similar expression levels under stress conditions in all tested genes (Figure 11). Transcript analysis revealed that expression of *NtCAT*, *NtSOD* and *NtAPX* genes increased significantly in transgrafted tobacco plants relative to WT/WT plants exposed to 10% PEG (Figure 11a–c). Expression of *NtERF* was also up-regulated by 1.9-, 1.8-, 2.4- and 2.2-folds in transgrafts N2/N2, WT/N2, D7/D7 and WT/D7, respectively, compared to WT/WT in response to OS (Figure 11d). Similarly, *NtRD29* showed 2-, 1.9-, 4.6- and 2.7-folds higher in N2/N2, WT/N2, D7/D7 and WT/D7, respectively, than WT/WT under stress conditions (Figure 11e). The expression of *NtERD1* followed the same response as *NtRD29* under OS with the highest induction of 6- and 4.6-folds in D7/D7 and WT/D7, respectively (Figure 11f). The expression of *NtMYC*, *NtHSP70* and *NtHSP26* also exhibited higher transcript levels in transgrafts than WT/WT under stress conditions (Figure 11g–i). Overall, the expression of the tested ORGs in the scion of N2/N2, WT/N2, D7/D7 and WT/D7 was significantly up-regulated compared to WT/WT after the PEG treatment, suggesting that transgenic rootstocks elevated ORGs expression in WT scion, improving its OS tolerance.

## 3. Discussion

### 3.1. Osmotic Stress Altered Expression Levels of Various ORG Genes

In this study, 21 ORGs were used to characterize the differences in their gene expression levels under conditions of OS using quantitative real-time RT-PCR (Figure 1). The tested genes are known to be involved in the abscisic acid (ABA) (i.e., *DREB*, *RD29*, *ERD7*, *ABI2* and *SNRK*), salicylic acid (SA) (i.e., *NPR1*) and jasmonic acid (JA) signaling pathways (i.e., *MYC2*, *MYB2*, and *MYB72*). In addition, the genes that induce different physiological responses under OS (i.e., *ADH1*, *STZ*, *UGT*, *ATPase*, *LIP2*, and *AFP4*) and six members of the heat shock protein (HSP) family (i.e., *HSP21*, *HSP26*, *5P*, *HSP17*, *HSP17-6C*, *HSP17-6A*, and *HSP18-1*) that have previously shown up-regulation under various stress conditions [52,53,54,55,56,57] were also tested. Numerous previous studies have shown that plants undergo changes in the level of expression of many stress-related genes depending on the nature and severity of stress conditions [58,59]. Changes in gene expression that occur following exposure to OS cause a series of physiological and biochemical alterations that contribute to increased OS tolerance. Here, we reported up-regulation in the transcription factors *DREB*, *RD29*, and *ERD7* under OS conditions. These genes are believed to be essential members inducing the transcription of other stress-inducible genes [60]. Previous studies also reported that *DREB* increases plant tolerance to freezing, drought, and high-salinity stresses when overexpressed in model plant species [61,62,63,64]. *RD29* has also been found to confer tolerance to different abiotic stresses when overexpressed in *Arabidopsis* [61] and tobacco [65]. Whereas ERD7 plays an important role in both ABA-dependent and ABA-independent signaling transduction pathways in response to abiotic stresses [66,67,68,69].

ORGs that have also been previously shown to play a key role in abiotic stress-related hormone signaling include *NPR1* and *MYB*. Our data showed that *NPR1* was significantly up-regulated after 6 h and 24 h of OS in the potato scion. It has been demonstrated that *StNPR1* gene expression increased after drought stress, suggesting an important role of *StNPR1* in plant’s response to drought stress [70]. The induction in *NPR1* levels can improve plant tolerance to OS through the positive regulation of salicylic acid (SA) as NPR1 is the SA receptor in the plant [71]. MYB transcription factors are also involved in plant responses to abiotic stress via the regulation of the phenylpropanoid pathway, which produces various secondary metabolic compounds involved in abiotic stress response [72] in addition to the regulation of various developmental processes in the stressed plant such as stomatal movement, the control of suberin and cuticular waxes synthesis, and crosstalk between different plant hormones [26]. Another gene family that is known to be involved in stress tolerance is the *HSP* family. These proteins can scavenge accumulated damaged proteins to maintain cellular homeostasis [73,74]. It has also been shown that HSP improve plant tolerance against abiotic stress by improving physiological processes such as membrane stability, photosynthesis, assimilate distribution, and the efficiency of water and nutrient use [52]. In the current study, the levels of five *HSP* family members significantly increased in potato in response to OS.

Another stress mitigation strategy that plants follow is to maintain a suitable energy balance during stress conditions via reducing growth by repressing the expression of several genes involved in photosynthesis and carbohydrate metabolism [51,75]. *STZ* has been demonstrated to be responsive to drought, salt, cold, and abscisic acid by reducing growth, saving plant resources for stress tolerance [75]. The increase in *UGT* and *ADH1* levels after PEG treatment may improve plant adaptation to OS through the biosynthesis and regulation of natural plant products and plant hormones [76] and the accumulation of osmolytes and callose [77], which in turn maintains cell homeostasis [78,79,80].

### 3.2. The Transport of NPR1 Transcripts through Graft-Union

The movement of mRNA over long distances has been confirmed in several plant species using the heterografting system [4,13,81,82,83,84,85]. In the present study, we confirmed the transport of the osmotic responsive gene *StNPR1* transcripts from rootstock to scion tissues under both normal and OS conditions (Supplementary Figure S2, Figure 2a,b). We also showed that the gene expression levels of potato *NPR1* in the tomato scion increased after exposure to stress compared to the heterograft control (Figure 2a)**,** indicating that OS may be a key factor in regulating the transport of *StNPR1* transcripts over long distances. Furthermore, we showed that gene homologs of the same family not necessarily share the same mRNA transport potential. Indeed, among the four members of the potato *NPR1*-related genes (*NIM1-1*, *NPR6A*, *NPR6B*, and *NPR1*), only the *NPR1* transcripts were detected in the scion of the heterograft.

The results shown in Figure 2a,b demonstrated that *StNPR1* has mobile mRNA. However, such mobility was not observed under a transgrafted system, in which WT tobacco scion was grafted to transgenic tobacco rootstocks overexpressing *StNPR1*. The mRNA mobility over long-distance is not well understood and is likely to be influenced by many factors. For instance, the lack of mRNA binding proteins may affect mRNA mobility. mRNA binding proteins have an essential role in mRNA movement and protect mRNA from degradation during the movement over long distances [15,16,86]. It has been suggested that the untranslated region (UTR) regions have conserved motifs that conjugate with mRNA binding protein to facilitate their movement [87,88]. In the present study, we used the coding region only of *StNPR1* for genetic transformation which could explain, at least partially, the lack of transcript mobility from the transgenic rootstock to non-transgenic scion. Further experiments need to be conducted to investigate the role of 5′ and 3′ UTRs of *NPR1* in the mobility and translation of its transcripts.

### 3.3. Overexpression of StNPR1 and StDREB1 Improved Tobacco Tolerance to Osmotic Stress

Although several osmotic-responsive genes were evaluated in the current study, our research focused on the characterization of *StNPR1* and *StDREB* that are involved in SA and ABA signaling pathways, respectively. NPR1 is a key regulator involved in plant response to biotic stress, and its regulatory mechanism has been relatively clear. The overexpression of *NPR1* in *Arabidopsis* results in various degrees of resistance to different biotic stresses [89]. Numerous studies have revealed that NPR1 is a key regulator of systemic acquired resistance (SAR), as *NPR1* works as a regulatory component that functions downstream of SA in the signal transduction cascade that mediates SAR induction [90]. Moreover, NPR1 is a master regulator of the salicylic acid (SA) signaling pathway as it works as a receptor for SA [71,91,92]. However, information about the role and the function of NPR1 in plant response to abiotic stress is largely unknown. *DREB*, on the other hand, is well known for its role in increasing tolerance to freezing, drought, and high salinity when overexpressed in model plant species [61,62,63,64,93]. In fact, *DREB* is a transcription factor that binds to the dehydration-responsive element (DRE)/C repeat (CRT), a *cis*-acting element that is involved in the up-regulation of several osmotic stress-related genes [60].

To explore the potential role of *StNPR1* and *StDREB1* in enhancing plant tolerance to OS, we overexpressed the coding region of *StNPR1* and *StDREB1* in tobacco using the constitutive CaMV35S promoter (Figure 4). The potential tolerance of the transgenic plants was initially assessed through leaf disc senescence assay (Figure 5) under NaCl (100 mM), mannitol (200 mM) and PEG (10%) stress conditions. The increase in EL and chlorophyll degradation, besides reducing cell viability has been confirmed to be a major markers of stress [94,95,96]. Under salt and OS, transgenic lines showed lower electrolyte leakage, more cell viability, and delayed chlorophyll degradation compared to control plants (WT and G3). This indicated that transgenic lines had better membrane permeability, lower oxidative stress and healthier leaf discs with higher photosynthetic capacity than the control group. These results are consistent with those reported in transgenic tobacco overexpressing apple *MdDREB76* [93]. Additionally, *SlNPR1* knock-out tomato plants have illustrated higher sensitivity to drought stress with higher H_2_O_2_, MDA and electrolytic leakage, suggesting that *NPR1* is essential for alleviating oxidative stress and cell membrane damage [70]. *NPR1* has also been reported to induce oxidative stress tolerance in *Arabidopsis* exposed to salt stress through *NPR1*-dependent SA signaling which was related to the control of Na^+^ flux in the roots and consequently its long-distance transport into the shoot under stress conditions [97].

N2 and D7 transgenic lines showed higher *GUS* gene expression which should also reflect the expression of the corresponding chimeric genes of *StNPR1* and *StDREB1*, respectively. Therefore, N2 and D7 were selected to examine the effect of different stress conditions on the morphology of the transgenic plants. The morphological changes were monitored under 100 mM NaCl, 200 mM mannitol and 10% PEG. N2, D7. Unsurprisingly, G3 plants expressing *GUS* only showed similar morphology to WT (Figure 6a). However, transgenic lines N2 and D7 had increased shoot length, fresh weight (FW), dry weight (DW) and root weight under different stress conditions compared to WT and G3. Previously, overexpression of *Syntrichia caninervis DREB8*, potato *DREB1* and tomato *DREB3* in *Arabidopsis*, potato and tobacco, respectively, increased root length and fresh weight and dry weight of transgenic seedlings growing under salt stress [95,98,99]. *NPR1* also plays an important role in controlling plant biomass under abiotic stress. Indeed, Arabidopsis *npr1* mutant, which lacks NPR1-dependent SA signaling, and plants overexpressing *NPR1*, have been tested under salt stress. The results showed higher fresh weight in plants overexpressing *NPR1* and lower fresh weight in *npr1* mutant growing under salt stress compared to wild-type *Arabidopsis* [97]. Our data also showed that overexpression of *StDREB1* significantly increased root weight in D7 seedling relative to WT and G3 under normal and OS conditions. Such change in root architecture could be attributed to ABA content that works as a negative regulator of lateral root formation under abiotic stress [26]. Interestingly, ABA levels in D7 plants were significantly lower than WT, under stress conditions.

### 3.4. The N2 and D7 Transgenic Rootstocks Improved the Growth Parameters of WT-Scions under Osmotic Stress

The effect of the tolerant transgenic rootstocks N2 and D7 on the WT-scions was examined under OS induced by PEG (10%) for 12 days. Relative water content and cell viability were significantly higher in transgrafted plants (N2/N2, WT/N2, D7/D7 and WT/D7), which alleviated stress injury, and thus, stress-induced damage was reduced compared to the control group (WT/WT, G3/G3 and WT/G3), whereas EL, which is a common stress marker, was significantly lower in scions of transgrafted plants compared to the scions of the control group indicating better membrane integrity [100]. Plants produce ROS under normal conditions during photosynthesis through electron transport chain in photosystem II (PSII) [101]. ROS also over-accumulate under abiotic stress, leading to oxidative stress conditions which in turn inhibits the PSII repair system [100,101]. High accumulation of ROS in the chloroplasts under stress conditions was proven to promote chlorophyll degradation, besides its negative effect on membrane permeability [100,102]. Here, it was observed that transgrafts with N2 and D7 rootstocks showed significantly higher chlorophyll content and reduced EL under OS compared to the control group. Control grafts (WT/WT, G3/G3 and WT/G3) also showed intense brown color precipitation after DAB staining indicating a higher level of H_2_O_2_ compared to transgrafted plants (N2/N2, WT/N2, D7/D7 and WT/D7) under OS. These observations indicate that *StNPR1* and *StDREB1* may be involved in alleviating oxidative stress. Indeed, our gene expression analyses showed significant up-regulation of tobacco genes encoding antioxidant enzymes such as superoxide dismutase (SOD), ascorbate peroxidase (APX) and catalase (CAT) in scion tissues grafted to transgenic N2 and D7 rootstocks, which could explain how these transgenes could contribute towards ROS homeostasis under OS [100]. Overall, our results demonstrate *StNPR1* and *StDREB1* transgenic rootstocks can modulate the physiological properties of the scion, increasing its tolerance to OS.

### 3.5. ABA Level in the Scion Is Rootstock Dependent

ABA is an essential stress-related hormone that is produced in plants to reduce the adverse effects of abiotic stress [34,101]. ABA is mainly produced in the root system and acts as a long-distance signal from the roots to the shoots to reduce stomatal conductance and consequently reduces transpirational water loss [3,34,36]. Li et al. (2018) [39] also reported that, under salt stress, the accumulation of ABA in tomato roots is partially regulated by shoot ABA export. The current study investigated the effect of tolerant transgenic rootstocks on ABA content in WT-scions under OS. ABA level was undetectable in all grafting combinations at zero time point when there was no stress. However, after 6 and 12 days of exposure to PEG, a marked increase in ABA content was observed in response to OS. Interestingly, the control plants showed around a 2-times increase in ABA content compared to N2/N2, WT/N2 at 12 days, but such an increase was not statistically significant. Additionally, ABA level was significantly lower in D7/D7 and WT/D7 than the control group at 12 days, suggesting that transgenic rootstocks overexpressing *StDREB1* negatively regulated the level of ABA in the leaves of WT-scion. Previous studies showed that the level of ABA in the rootstock regulated ABA content in the scion [34,39,41], which in turn is associated with gas exchange, stomatal conductance and water status [103]. The lower ABA content in D7/D7 and WT/D7 indicated that *StDREB1* might improve OS tolerance in tobacco through an ABA-independent signaling pathway. Along the same vein, overexpressing *OsDREB6* and *LlDREB1G* was found to enhance tolerance to abiotic stress in ABA-independent signaling pathway under different abiotic stresses [104,105]. We also found that the leaf content of ABA in the control grafts significantly increased from 6 to 12 days in WT/WT, G3/G3 and WT/G3; which in turn may have reduced stomatal conductance, gas exchange, consequently reducing photosynthetic rate [39,99]. On the other hand, the notable increase in ABA content from 6 to 12 days in N2/N2, WT/N2, D7/D7 and WT/D7 was insignificant and still lower than the control plants. This suggested that at six days, the plants might have acquired OS tolerance to maintain physiological processes using a different strategy to maintain water status, such as improved root system and/or higher osmolytes content, than the continuous increase in ABA and enhanced stomatal closure to reduce water loss, thereby lowering the photosynthesis which may promote the onset of senescence [99]. It has also been found that the increase in rootstock vigor is correlated with the reduction in ABA concentrations. Indeed, rootstocks with higher ABA levels showed early senescing and decreased net photosynthesis [41]. Overall, ABA is one of the most important long-distance signals in controlling plant growth under abiotic stress by regulating photosynthesis and transpiration, although the involvement of other factors cannot be ruled out and remains to be investigated.

### 3.6. Transgenic Rootstocks Improve WT-Scion via Up-Regulation of Various ORGs

This study investigated the influence of the transgenic rootstocks on gene expression regulation in the WT-scion. As mentioned above, the transcripts of tobacco *NtSOD*, *NtCAT* and *NtAPX* genes were increased by OS in WT-scions grafted on N2 and D7 compared to control plants. The increased gene expression of *CAT*, *SOD* and *APX* was reported in previous transgenic studies where *MdDREB76* [93] and *SbUSP* [96] overexpressed in tobacco enhanced plant tolerance to salt, drought and OS. The gene expression levels of tobacco *NtERF, NtRD29* and *NtERD1* were also found to be up-regulated in the scion of transgrafted plants relative to the control plants. Indeed, the transcript levels of these genes were almost 2–3 folds higher in D7/D7 and WT/D7 compared to N2/N2 and WT/N2. This increase might result from *RD29A*, *ERD1* and *ERF* being major abiotic stress-related genes that act downstream *DREB* signaling [62]. Previous studies reported that overexpressing of *MdDREB76* and *ScDREB8* in tobacco and *Arabidopsis*, respectively, elevated the gene expression of *ERF*, *ERD1*, *ERD10A ERD10D*, conferring salt and drought tolerance to the transgenic plants [93,98]. The expression of *MYC2* was also highly regulated in WT-scions grafted on N2 and D7 compared to control group under OS (Figure 11g). It has been found that MYB transcription factor plays a key role in ABA signaling pathway [81] through the induction of stress-related genes such as RD22. MYB has also been reported to be involved in stomatal conductance under stress conditions, which in turn controls water status in plants [26,106]. The tomato *Slnpr1* loss of function mutant has shown a lower drought tolerance that was associated with the downregulation of various drought-related genes, including *DREB,* suggesting that *NPR1* might be involved in ABA signaling pathway under drought stress [70]. This might explain the up-regulation of *NtERF*, *NtRD29*, *NtERD1* and *MYC* genes in the scion of N2/N2 and WT/N2 compared to the control plants. Heat shock proteins (HSP) are a large group of transcription factors that regulate protein transport, folding and maintenance of correct protein structure to protect cells from abiotic stress [107]. In the current study, the up-regulation of *NtHSP70* and *NtHSP26* was observed in transgrafted plants relative to the control plants (Figure 11h,i). Likewise, the overexpression of *MdDREB76*, *LlDREB1G* and *ScDREB8* elevated the transcript level of *NtHSP70* and *NtHSP26* in tobacco and *Arabidopsis* plants growing under different abiotic stress conditions imparting stress tolerance to the transgenic plants [93,98,105]. Additionally, SA is involved in the induction of various genes encoding heat shock proteins (HSPs) and chaperones [108]. In fact, *Arabidopsis NPR1* transcripts are up-regulated in response to low temperatures and NPR1 protein interacts with heat shock transcription factor 1 (HSFA1) to induce the expression of *HSFA1*-related genes under cold stress including *HSP70* in *Arabidopsis* [109]. Overall, the results of the current study indicated that transgenic rootstocks overexpressing *StNPR1* and *StDEB1* confer OS tolerance to the WT-scion through up-regulation of genes encoding transcription factors and major enzymes that alleviate oxidative stress and modulate physiological processes, resulting in a better plant performance under stress conditions compared to WT/WT plants.

## 4. Conclusions

The identification of several ORGs that are highly up-regulated in potato following OS indicates that various signaling molecules are involved in improving OS tolerance in potato plants. Although we found that *StNPR1* transcripts were transported from the potato rootstock to tomato scion, mRNA of *StNPR1* was not transported from the transgenic rootstock to the wild-type scion in transgrafted tobacco. Our results also revealed that tolerant rootstocks overexpressing *StNPR1* and *StDREB1* enhanced the growth and performance of wild-type scions in transgrafted tobacco by activating various osmotic responsive genes. These genes encode for enzymes and transcription factors and that alleviate oxidative stress and modulate physiological properties of the scion, including RWC, EL, CV, chlorophyll and H_2_O_2_ contents, thereby improving OS tolerance in transgrafted tobacco plants. Together, the results of the present study elucidated, at least partially, the underlying mechanisms of the induced defense response in WT-scions grafted to transgenic rootstocks in osmotically stressed transgrafted tobacco. Our study also paves the way for incorporating *StNPR1* and *StDREB1* as two potential candidates for producing stress-resilient crops through the transgrafting technology, which could have major implications in the horticulture industry.

## 5. Materials and Methods

### 5.1. Grafting, Transgrafting and Growth Conditions

Four-week-old tomato seedlings and eight-week-old potato seedlings with uniform stem diameter (around 5 mm) were used as the scion and rootstock, respectively, to produce a heterograft using cleft grafting method modified from Notaguchi et al. (2012) [8]. In brief, a clear sharp wedge was made by a sharp blade at the bottom of the scion, and then it was inserted into a V-shaped slit made at the top of the rootstock (10–15 cm). All the grafted plants were grown in the mist bed for five days. The tomato and potato grafted plants were transferred to a greenhouse which had light intensity of 300 μmol m^−2^ s^−1^, a day and a night-time temperature of 21 ± 2 °C and a diurnal cycle of 16 h light/8 h dark. The F_2_ seeds of G3, N2 and D7 were germinated on MS [110] selection medium with vitamins (Phyto Technology laboratories, Shawnee Mission, KS, USA) and containing 20 μg/mL hygromycin (Goldbio, St. Louis, MO, USA) and 300 µg/mL timentin (PhytoTechnology Laboratories, Shawnee Mission, KS, USA) for two weeks while WT seeds were germinated on the same medium without the antibiotics. The seedlings which survived were subcultured on the selection medium for 5 weeks before transferring the plants into the growth chamber. Transgrafting was performed using 5 weeks old tobacco plants following the same procedure as described above to create six transgrafting treatments and wild-type homograft. All grafted tobacco plants were grown in a growth chamber with light intensity of 200 μmol m^−2^ s^−1^, a constant temperature of 23 ± 2 °C and 16 h light.

### 5.2. Plant Materials and Sample Collection for Analysis of mRNA Transport

Tomato seeds (*Solanum lycopersicum* ‘Beefsteak’) were germinated and grown in a propagation mix in a greenhouse. Four-week-old potato rooted microshoots (*Solanum tuberosum* ‘Gold Rush’) were transferred from an in vitro culture to a mist bed for five days and then were moved to the same growth conditions as the tomato seedlings. Experiments were performed using the tomato/potato heterograft (T/P), potato/potato homograft (P/P) as the positive control and tomato/tomato homograft (T/T) as the negative control. Volume of 200 mL/plant of 30% polyethylene glycol (PEG 6000) (Sigma-Aldrich, St. Louis, MO, USA) was used to induce OS; the same amount of water was added to the control. The sampling of the scion was performed 6 h after inducing stress by taking 5 cm of the scion’s stem (1 cm above the graft union). All the samples were flash-frozen in liquid nitrogen before storing them at −80 °C for further use. Three reference genes (*GTP-binding protein SAR1A* (*GTP*), *TIP41-like family protein* (*TIP*), and *Clathrin adaptor complexes* (*Clathrin*)), which have shown highly stable expression levels in tomato [111], were used in the present study. To make sure that the normalized relative gene expression of the potato genes with mobile transcripts in tomato scion of the heterograft is normalized based on tomato transcripts only and not other mobile transcripts from potato rootstock, the movement of the housekeeping genes was determined using specific potato primers (*StGTP*, *StTIP*, and *StClathrin*), which are presented in Supplementary Table S1.

### 5.3. Tobacco Transformation and Molecular Confirmation of Transgenics

The constructs of pCambia1301:*StNPR1* and pCambia1301:*StDREB1* were synthesized by Biomatik, USA (http://www.biomatik.com), accessed on 15 May 2018. Constructs containing a hygromycin resistance selectable marker, regulated by CaMV35S enhanced promoter and the transformed genes downstream of another CaMV 35S promoter, were introduced into tobacco (*Nicotiana tabacum*) plants through *Agrobacterium tumefaciens* (EHA105)-mediated leaf disc method [112,113]. The regenerated transformants (F_1_) were screened by growing on a Murashige and Skoog MS+vitamins selection medium supplemented with 30 μg/mL hygromycin and 300 µg/mL Timentin, 3% sucrose, and 0.70% (w/v) agar (Fisher Chemical, Fair Lawn, NJ Belgium). The pH was adjusted to 5.7. The transgene’s presence was confirmed by PCR amplification using genomic DNA as a template in the wild type (WT), G3 (the transgenic line overexpressing the empty vector with *GUS*), N2 and N4 (the transgenic lines over-expressing *StNPR1*) and D1, D7, and D11 (the transgenic lines over-expressing *StDREB1*). *NtActin* was used as a reference gene. The relative gene expression of *GUS* was analyzed in transgenic lines by qRT-PCR with three biological and three technical replicates. Elongation factor-1α (*NtEF1*) gene was used as the internal control.

### 5.4. Analyses of F_1_ and F_2_ Transgenic Plants Exposed to Abiotic Stresses

The stress tolerance capacity of the transgenic lines was initially tested using leaf disc assay. Leaf disc assay was performed using leaves of six weeks old F_1_ plants modified from Singla-Pareek et al. (2003) [114]. The 2 cm diameter leaf discs of transgenic lines (G3, N2, N4, D1, D7 and D11) and WT were punched out and floated on water supplemented with 100 mM sodium chloride (NaCl) (Fisher Scientific, Fair Lawn, NJ, USA), 200 mM mannitol (Phyto Technology laboratories, Shawnee Mission, KS, USA) and 10% PEG for 9 days. The experiment was maintained on the bench at room temperature, and phenotypic changes were monitored over 9 days. The effect of stress treatments was evaluated by visually analyzing phenotypic changes among leaf discs along with determination of physiological parameters including electrolyte leakage (EL), cell viability (CV), and chlorophyll content.

The F_2_ seeds of G3, N2 and D7 were germinated on MS+vitamins selection medium containing 20 μg/mL hygromycin and 300 µg/mL timentin for two weeks while WT seeds were germinated on MS+vitamins for the same duration. The surviving seedlings were subcultured on MS+Vitamins selection media supplemented with 100 mM NaCl, 200 mM mannitol and 10% PEG and incubated for four weeks under controlled environment conditions. The transgenic lines were analyzed for shoot length, root length, fresh weight (FW), dry weight (DW) and root fresh weight.

### 5.5. Stress Tolerance of Transgrafted Tobacco Plants

To assess transgraft tolerance to OS, all the grafts were drenched with 10% PEG solution after one month of grafting. The grafting treatments were: wild type homograft, i.e., wild type scion grafted on wild type rootstock (WT/WT), G3 scion grafted to G3 rootstock (G3/G3), WT scion grafted on G3 rootstock (WT/G3), N2 scion grafted on N2 rootstock (N2/N2), WT scion grafted on N2 rootstock (WT/N2), D7 scion grafted on D7 rootstock (D7/D7) and WT scion grafted on D7 rootstock (WT/D7). Each treatment contained 12 grafts divided into three biological replicates. Leaf samples were collected at zero-day (control), 3, 6, 9 and 12 days after treatment for physiological, biochemical and molecular analyses.

### 5.6. Measurement of Electrolyte Leakage, Cell Viability, Relative Water Content and Chlorophyll Content

The injuries in the cell membrane were assessed by estimating the electrolyte leakage using an electrical conductivity meter, as per Bajji et al. (2002) and Sharma et al. (2019) [93,115]. Fresh leaves were cut into 0.5 cm discs, six-leaf discs were washed with deionized water, then immersed in 20 mL of deionized water and kept on the shaker at 100 rpm at room temperature. Initial electrical conductivity (IEC) was recorded after 24 (h). The samples were then autoclaved at 121 °C for 20 min to release all the electrolytes. After cooling down the samples to room temperature, the final electrical conductivity (FEC) was measured. Electrolyte leakage was calculated by the formula: EL (%) = (IEC/FEC) × 100. Cell viability was estimated by 2,3,5-triphenyltetrazolium chloride (TTC) (Sigma-Aldrich, St. Louis, MO, USA) assay [116]. Four 0.5 cm leaf discs were incubated in 0.1% TTC at 37 °C in the dark for 6 h. Samples were then incubated in 1 mL of 95% ethanol (Greenfield Global, Brampton, ON, Canada) at 60 °C for 5 min. The absorbance of formazan was recorded at 470 nm using a plate reader (Biotech, USA). Cell viability was determined using the formula: CV (%) = (1− (OD_0_ − OD_T_ /OD_0_))*100, where OD_0_ is the OD of the sample at zero time and OD_T_ is the OD of the same treatment after stress treatment. The relative water content (RWC) was recorded as described by Aneja et al. (2015) [55], with minor modifications. The fresh weight (FW) of leaf segments was immediately measured. Then, the leaves were soaked in distilled water at room temperature for 24 h, the turgid weight (TW) was recorded. The leaves were dried in the oven for 24 h at 70 °C, and the dry weight (DW) was recorded. The RWC was calculated using the formula: RWC (%) = (FW − DW)/(TW − DW) × 100. Total chlorophyll was determined through immersing 50 mg from the fresh leaves in ethanol 95% and incubation at 70 °C for 5 min. Then, the absorbance was measured at room temperature using a plate reader (Biotech, USA). Total chlorophyll amount was determined as described by Lichtenthaler (1987) [117].

### 5.7. In Situ Localization and Estimation of Hydrogen Peroxide (H_2_O_2_)

The accumulation of H_2_O_2_ was detected through the histochemical staining of the leaves with 3,3-diaminobenzidine (DAB) (Sigma-Aldrich St. Louis, MO, USA) method, modified from Daudi and O’Brien (2012) [118]. Leaves were immersed in DAB solution in the dark on the shaker at 100 rpm for 5 h. The chlorophyll was bleached by incubating the leaves with ethanol at 90 °C for 5 min. The H_2_O_2_ concentration was determined as described earlier by Patterson et al. (1984) [119]. Then, 50 mg of ground leaves of the scions were extracted in 500 µL of precooled acetone and centrifuged for 10 min at 1500× *g*. Titanium chloride (Sigma-Aldrich St. Louis, MO, USA) (2% *v/v* of the extracted supernatant) and concentrated ammonia (100 µL) were added to the supernatant prior to centrifuging the reaction mixture at 1500× *g* for 10 min. Absorbance was read at 410 nm, and the H_2_O_2_ concentration was calculated according to the standard curve for peroxide determination.

### 5.8. Extraction and Analysis of ABA

ABA content in tomato and potato was extracted and analyzed using an LC-MS method adopted from Ayyanath et al. (2021) [120]. In brief, leaf samples were ground in liquid nitrogen to a fine powder. Then, 150 mg of each sample was extracted with 500 μL extraction solution (50% MeOH (Thermo Scientific, Fair Lawn, NJ, USA) and 4% acetic acid (Fisher Scientific, Mississauga, ON, Canada) in Milli-Q water), and the samples were sonicated on ice for 30 min prior to centrifugation for 2 min at 13,000 rpm. Subsequently, the supernatant was dispensed into a fresh tube and diluted 5× in 10 mM ammonium acetate (Sigma Aldrich, Mississauga, ON, Canada) (pH 9; adjusted with ammonium hydroxide (Sigma Aldrich, Mississauga, ON, Canada)). Samples (500 µL each) were filtered-centrifuged using 0.22 µm Millipore tubes and the supernatant was used for quantification using ultra-performance liquid chromatography (ULPC) as described by Erland et al. (2017) [121]. Aliquotes of 3 µL were injected onto a Waters Acquity BEH Column (2.1 × 50 mm, i.d. 2.1 mm, 1.7 µm) on a Waters Acquity Classic UPLC system with detection using an Aquity QD single quadrupole mass spectrometer (MS) controlled by Empower 3 (Waters, Mississauga, ON, Canada). Samples were run on a gradient with A-10 mM ammonium acetate pH 9, adjusted with ammonium hydroxide; B-100% MeOH with initial conditions of 95% A 5% B increased to 5% A 95% B over 4.5 min using an Empower curve of 8. Column temperature was 40 °C and flow rate was 0.5 mL/min. Capillary voltage was 0.8 kV, and probe temperature was 500 °C with a gain of five. ABA was monitored in single ion recording mode and quantified in ng.g^−1^ FW using the standard curve.

ABA levels in tobacco leaves were also determined using the methods described by Ayyanath et al. (2021) [120] with some modifications. In brief, leaf samples were ground in liquid nitrogen to a fine powder. Then, 150 mg of each sample was extracted with 500 μL extraction solution (10 mM ammonium acetate pH 9.0) vortexed for 1 min at maximum speed. Then, 100 μL 5% acetic acid (glacial, Fisher Scientific, Mississauga, ON, Canada) was added and vortexed for a further 1 min at the maximum speed. Samples were centrifuged at 14,000× *g*, at 4°C for 15 min. The supernatant was used for analysis following Erland et al. (2017) [121] with some modifications. Samples (5 µL each) were injected onto a Waters Acquity BEH Column (C18, 1.7 μm, 2.1 × 50 mm) on a Waters Acquity Classic ultra-performance liquid chromatography (UPLC) system with detection using an Aquity QDa single quadrupole mass spectrometer (MS) controlled by Empower 3 (Waters, Canada). Samples were run on a gradient with A-0.1% Formic Acid (Thermo Scientific, Fair Lawn, NJ, USA); B-100% Acetonitrile (Thermo Scientific, Fair Lawn, NJ, USA) with initial conditions of 97% A and 3% B increased to 3% A 97% B for 4.5 min, then 97% A and 3% B at 4.6 min. Column temperature was 40 °C, autosampler temperature was 4.0 °C and flow rate was 0.5 mL/min. ABA was monitored in single ion recording mode and quantified in µg.g^−1^ FW using standard curve. Capillary voltage was 0.8 kV and probe temperature was 500 °C with a gain of five.

### 5.9. DNA and RNA Extraction and Expression Analysis of Abiotic Stress-Responsive Genes

Tissue samples were ground to a fine powder in liquid nitrogen, after which total RNA was extracted using CTAB [122]. cDNA syntheses were carried out from 2000 ng (from tomato and potato) and 2500 ng (from tobacco) of purified RNA in a 20 µL reverse transcription reaction mixture using a High-Capacity cDNA Reverse Transcription Kit (Applied Biosystems, Vilnius, Lithuania) following the manufacturer’s instructions. The cDNA was diluted 1/10 with ultrapure water. DNA was extracted using a DNA extraction kit (QIAGEN, Hilden, Germany) following the manufacturer’s instructions. The expression analysis of the selected osmotic responsive genes was quantified through quantitative real-time PCR (qRT-PCR) with three biological and three technical replicates for each sample along with negative control. The qRT-PCR reaction was performed in a 10 µL reaction volume. The mixture contained 2.5 µL of cDNA, 1X SsoFast^TM^ EvaGreen^®^ Supermix (Bio-Rad, California, CA, USA) and 0.4 µM of each primer. The qRT-PCR reactions were carried out using CFX Connect^TM^ Real-Time System (Bio-Rad, Singapore) for one denaturation cycle at 95 °C for 30 s then 40 cycles of 95 °C for 10 s (denaturation), followed by 60 °C for 20 s (annealing and extension). Normalized relative fold expression was calculated using the 2^−∆∆CT^ method and 2^−∆CT^ method was used to calculate relative gene expression. Semi-quantitative-reverse transcription (RT-PCR) analysis was performed using Platinum^®^ PCR SuperMix to amplify 613 bp, 501 bp of *Actin* and *StNPR1*, respectively, (Figure 2b) and 432bp, 524 bp, 501bp and 534bp of *NtActin*, *GUS*, *StNPR1* and *StDERB1*, respectively, in transgenic tobacco plants (Figure 4b) after 36 cycles. The primers used for gene expression analysis are shown in Supplementary Tables S1 and S2.

### 5.10. Statistical Analysis

All the plants were arranged in a complete randomized design (CRD). An analysis of variance (ANOVA) was performed using GraphPad Prism v9. Least square means of three independent biological replicates were compared using Dunnett or Tukey–Kramer test with α = 0.05 level. Test of normality was performed prior to any analysis, and the log model was used to transfer the date from non-Gaussian to Gaussian when required. The means ± standard error of mean responses of each parameter of the three replications were presented in the graphs. The results were confirmed by repeating the experiments twice.

## Data Availability

All data generated or analyzed during this study are included in this published article and its Supplementary Information Files.

## Supplementary Materials

The following are available online at https://www.mdpi.com/article/10.3390/ijms22168398/s1, Figure S1: Transport of housekeeping mRNAs from the potato rootstock to the tomato scion 6 h after exposure to osmotic stress, Figure S2: Transport of ORG mRNAs from the potato rootstock to the tomato scion 6 h after exposure to osmotic stress, assessed by qRT-PCR, Table S1: Sequence of primers used to assess expression of osmotic related genes in potato and tomato homo and heterografts, Table S2: Sequence of used primers in tobacco experiment.

## Abbreviations

OS	Osmotic stress
ORGs	Osmotic-responsive genes
PEG	Polyethylene glycol
SA	Salicylic acid
ABA	Abscisic acid
HSP	Heat shock protein
NPR1	non-expressor of pathogenesis-related (PR) gene 1
DREB	Dehydration responsive element binding
WT	Wild type
CV	Cell viability
EL	Electrolyte leakage
RWC	Relative water content
ROS	Reactive oxygen species
H_2_O_2_	Hydrogen peroxide
