# Supplementary material for: Rootstocks Overexpressing StNPR1 and StDREB1 Improve Osmotic Stress Tolerance of Wild-Type Scion in Transgrafted Tobacco Plants
Source: Int J Mol Sci. 2021 Aug 5;22(16):8398. doi: 10.3390/ijms22168398 (PMC8395105; doi:10.3390/ijms22168398)
Supplement: Supplementary file 1 [file ijms-22-08398-s001.zip › ijms-1290063-supplementary.pdf]

Rootstocks Overexpressing *StNPR1* and *StDREB1* Improve Osmotic Stress Tolerance of Wild-Type Scion in Transgrafted Tobacco Plants

Supplementary materials

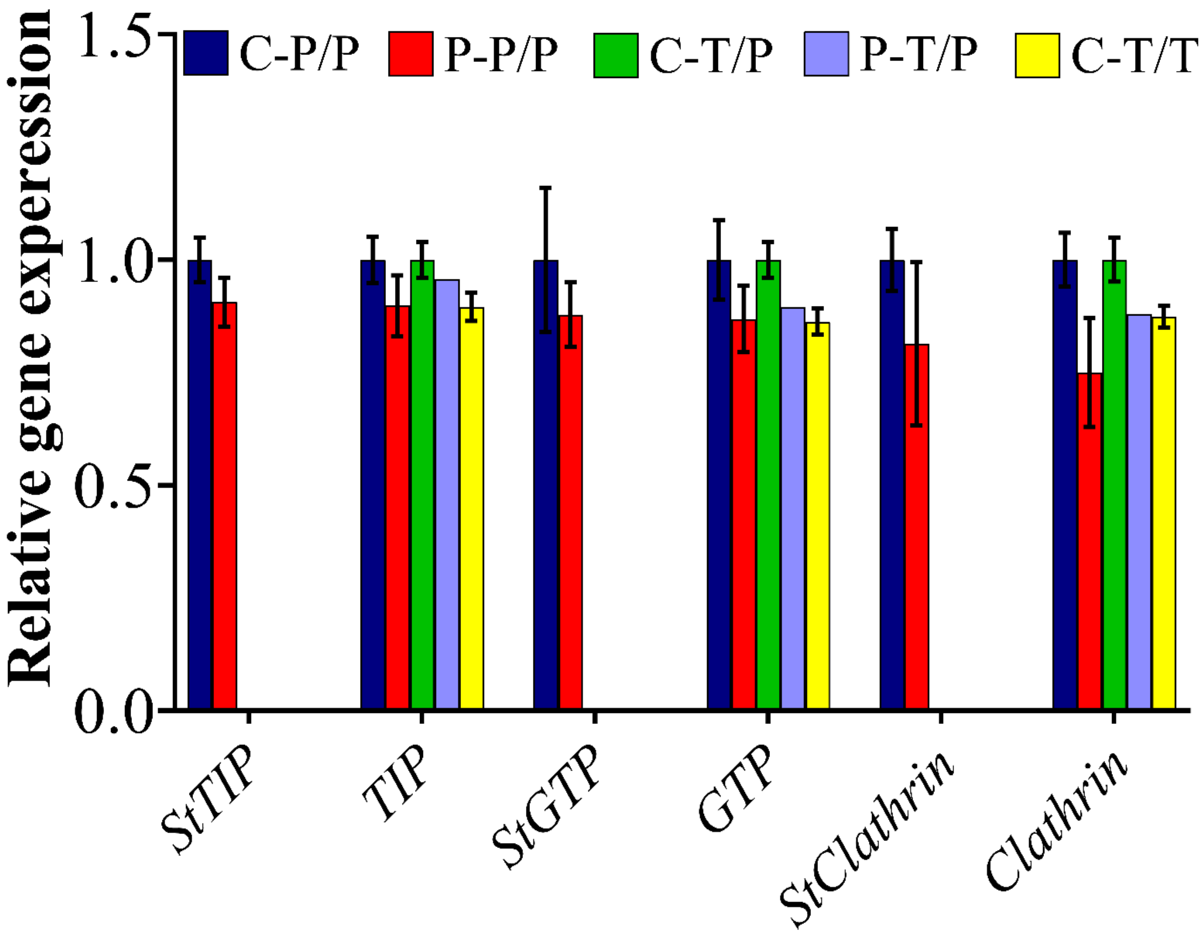

Figure S1. Transport of housekeeping mRNAs from the potato rootstock to the tomato scion 6 h after exposure to osmotic stress. Control-potato homograft (C-P/P), PEG-potato homograft (P-P/P), control-tomato/potato heterograft (C-T/P), PEG-tomato/potato heterograft (P-T/P) and control tomato homograft (C-T/T). The relative expression of potato genes in P-P/P was calculated relative to the control (C-P/P). The relative expression of tomato genes in tomato scions was calculated relative to the control (C-T/T). The values are the mean of three biological replicates. *StGTP – StTIP – StClathrin* are results of specific potato primers used to test the transport of the housekeeping genes' transcripts and *GTP – TIP – Clathrin* results of primers used to amplify both potato and tomato transcripts to select the most consistent reference gene.

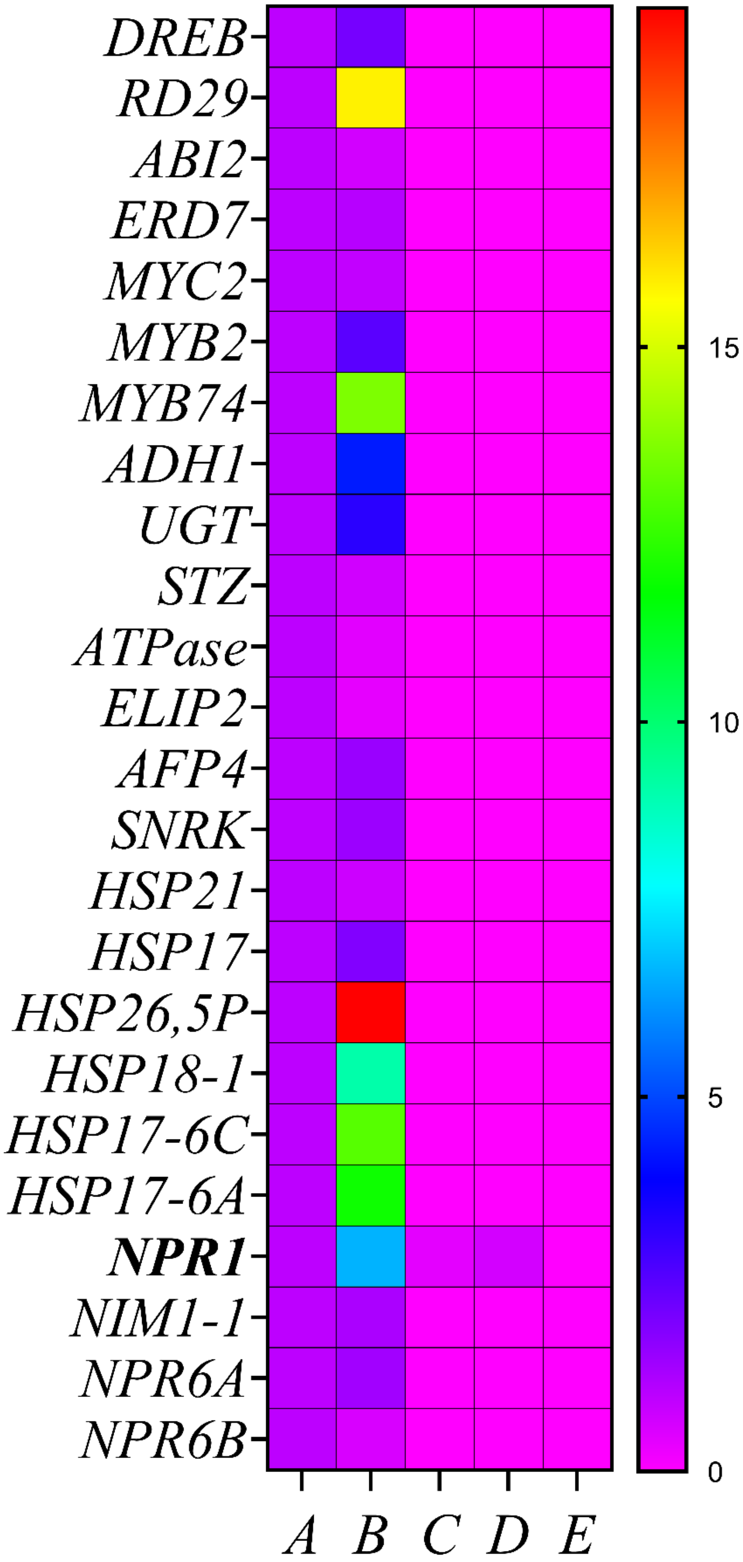

DREB
RD29
ABI2
ERD7
MYC2
MYB2
MYB74
ADH1
UGT
STZ
ATPase
ELIP2
AFP4
SNRK
HSP21
HSP17
HSP26,5P
HSP18-1
HSP17-6C
HSP17-6A
NPR1
NIM1-1
NPR6A
NPR6B
A
B
C
D
E
15
10
5
0

Figure S2. Heat map shows the transport of ORG mRNAs from the potato rootstock to the tomato scion 6 h after exposure to osmotic stress, assessed by qRT-PCR. Plants were osmotically stressed by watering with 30% PEG (6000) four weeks after graft establishment. Tissues samples located 5 cm above the graft union were collected from untreated plants (control) and 6 h of PEG-treated plants. A represents control-potato homograft (C-P/P), B represents PEG-potato homograft (P-P/P), C represents control-tomato/potato heterograft (C-T/P), D represents PEG-tomato/potato heterograft (P-T/P) and E represents control tomato homograft (C-T/T). The relative expression of potato genes was calculated relative to the control (C-P/P), and normalized to TIP. The bars show the normalized relative expression of potato ORG. Values were obtained from the means ± SEM, n = 3. The purple color means that the transcripts were not detected in the scion of C, D and E for all tested ORG except for NPR1, potato transcripts were not detected in control tomato homograft only.

Table S1. Sequence of primers used to assess expression of osmotic related genes in potato and tomato homo and heterografts.

Accession #	Gene Name	Primer sequence
**Primers used for qRT-PCR**		
PGSC0003DMT400068136	*Vacular H+-ATPase A2 - F*	AAATGGGAAGGTGCTTGG
PGSC0003DMT400068136	*Vacular H+-ATPase A2 - R*	TCGAGGACAACAAATTCCAG
PGSC0003DMT400037083	*LTI65 – F*	GGGAAAAGTGATCAGGATGC
PGSC0003DMT400037083	*LTI65 – R*	TTTGAAGGGACGGACTCAA
PGSC0003DMT40003201	*DREB – F*	CCCCTGAAATGGCTGCAAG
PGSC0003DMT40003201	*DREB – R*	CGAGGCAACGATTCAACGA
PGSC0003DMT400045204	*MYC2 – F*	CGTCGAGGGCTTTGAATTA
PGSC0003DMT400045204	*MYC2 – R*	GGCGTACGTAGCTGCAAAT
PGSC0003DMT400022600	*MYB2 – F*	AGCTTTTGGGGAGAGAGACA
PGSC0003DMT400022600	*MYB2 – R*	TTAACCCAACCCAATTCCA
PGSC0003DMT400057690	*MYB 74 – F*	GTAACCAAATTCAAGCCCCTA
PGSC0003DMT400057690	*MYB 74 -R*	AAATGTTGAACCATCGGATGT
PGSC0003DMT400066189	*St-TIP4 1 -F*	CTTGGGAAGAGGTTGCTGGT
PGSC0003DMT400066189	*St-TIP4 1 – R*	TGGGTTCTGTGTCGTTCATT
PGSC0003DMT400052825	*St-Clathrin – F*	GTTTGGAAATGAGGCAAAG
PGSC0003DMT400052825	*St-Clathrin – R*	CATTTCCAGGAACCAGACAA

PGSC0003DMT400023327	*St-GTPase – F*	TTCCCGTCTGGAAAAGATGG
PGSC0003DMT400023327	*St-GTPase – R*	TGCTGAAGAAGAAGCGGATAA
PGSC0003DMT400066189	*Sl & St-TIP4 1 -F*	GTTGGGAGATCGAGTGTCG
PGSC0003DMT400066189	*Sl & St-TIP4 1 – R*	CATCTCCGGCAAGTGAGTT
PGSC0003DMT400052825	*Sl & St-Clathrin – F*	TGCTTCCTTTCTGGAATGC
PGSC0003DMT400052825	*Sl & St-Clathrin – R*	GGACGGGACTTGAGTTGTG
PGSC0003DMT400023327	*Sl & St-GTPase – F*	AGCGACTGTTCCCTTCCTC
PGSC0003DMT400023327	*Sl & St-GTPase – R*	CCCAAGGTGATAACGCAAC
PGSC0003DMT400037120	*CRT binding factor 2A – F*	CTTCCGACCATCATCATCAGA
PGSC0003DMT400037120	*CRT binding factor 2A – R*	GGAGGAGGTAGCATGAGTCC
PGSC0003DMT400061367	*ERD7 – F*	CACTGAACTTGTAAACTGGTA
PGSC0003DMT400061367	*ERD7 – R*	AAACTATGACTGACGTGAAC
PGSC0003DMT400002412	*NPR1 – F*	TCCACCAGCTCACTCCTCTG
PGSC0003DMT400002412	*NPR1 – R*	ACAAATCACCAAAGCCACAA
PGSC0003DMT400075333	*ABI1B – F*	GGTTCTTGCTGTTGTGGCTA
PGSC0003DMT400075333	*ABI1B – R*	CGCCCCATTAATCAGGATAC
PGSC0003DMT400045362	*UGT73E2 – F*	GACTAACAATGGGTGTTCCTA
PGSC0003DMT400045362	*UGT73E2 – R*	AGTAATCGCGCAATGTCAA
PGSC0003DMT400016494	*ELIP2 – F*	GCCAAACCAAGTGATGGAA
PGSC0003DMT400016494	*ELIP2 – R*	AACCCAGAAGACTACGCTGT
PGSC0003DMT400063937	*ADH1 – F*	TGCGTATTTGGCATAATTGC
PGSC0003DMT400063937	*ADH1 – R*	GCACCGTCAGTATTGTGTGA
PGSC0003DMT400008351	*HSP21 – F*	AATACTCCATCCCAGTTCCAA
PGSC0003DMT400008351	*HSP21 – R*	AAGCAATCTTCACACTGGAG
PGSC0003DMT400078007	*HSP17,6 – F*	AAGGAATTAGGCTTTCCAGGTT
PGSC0003DMT400078007	*HSP17,6 – R*	CCATTAAGCAGAAACAGAGCA
PGSC0003DMT400055958	*HSP17,6A – F*	GAAGCTCCTCGAAACTTTCCT
PGSC0003DMT400055958	*HSP17,6A – R*	CTTGGATGTTGTGATTTCGTG
PGSC0003DMT400078007	*HSP17,6C -+ F*	CTTCAAGGAATTAGGCTTTCC

PGSC0003DMT400078007	*HSP17,6C – R*	GCAGAAACAGAGCAGACTCAAG
PGSC0003DMT400054660	*NIM1-1 – F*	ACACGTGGGTCCACTTTGT
PGSC0003DMT400054660	*NIM1-1 – R*	GAACAACATTCATTCCAGTC
PGSC0003DMT400021076	*NPR6A – F*	GGTGCAAATGTAGCAACGACT
PGSC0003DMT400021076	*NPR6A – R*	TCAAAAAGGGGTAGAGGCAA
PGSC0003DMT400027591	*NPR6B – F*	TCCATATTGCTCCACCCTCT
PGSC0003DMT400027591	*NPR6B – R*	CGGCCCACAAAAGAATTTCC
**Primers used for RT- PCR**		
PGSC0003DMT400002412	*RT-PCR-NPR1-F*	GGATTTGCTTTGCTTGCTG
PGSC0003DMT400002412	*RT-PCR-NPR1-R*	ACCTCGACCGGATCATTG
PGSC0003DMT400060953	*RT-PCR-Actin2-F*	GGCAGAAGGTGAGGATATT
PGSC0003DMT400060953	*RT-PCR-Actin2-R*	TGTGGTGGTGAAAGAGTAG

Table S2. Sequence of used primers in tobacco experiment.

**Primers used for qRTPCR**		
**Accession #**	**Gene Name**	**Forward primer**
AF234297.1	*GUS-F*	GCTGATAGCGCGTGACAAA
AF234297.1	*GUS-R*	CGCGAAATATTCCCGTGCA
NM_001326165.1	*NtEF1-F*	GGACGTTTTGCTGTGAGGG
NM_001326165.1	*NtEF1-R*	GTGACCTTGGCACCAGTTG
EU580435.1	*NtSOD-F*	AGCTACATGACGCCATTTCC
EU580435.1	*NtSOD-R*	CCCTGTAAAGCAGCACCTTC
U07627.1	*NtCAT-F*	TGCCATTGATGCCAGTTGG
U07627.1	*NtCAT-R*	CAGGGTTAAACGCGAGCTG
D85912	*NtAPX-F*	CAAATGTAAGAGGAAACTCAGAGGA
D85912	*NtAPX-R*	CAGCCTTGAGCCTCATGGTACCG
JX682668.1	*NtERF-F*	TTGGCACCTTTGACACTGC
JX682668.1	*NtERF-R*	GATGGGGGAAGTTGAGCCT

GU144573.1	*NtERD1-F*	GGTGTCAATGGAATTCCGCA
GU144573.1	*NtERD1-R*	ACGGGGGATCTTGGTGAAC
HE653924.1	*NtMYC-F*	ATGGGGTGCAGATGACCAA
HE653924.1	*NtMYC-R*	GGCAGGGCTTTGGTGTTAC
AB689673.1	*NtHSP70-F*	GACTGTGTTGATCCCCCGA
AB689673.1	*NtHSP70-R*	CAGGCTGGTTGTCCGAGTA
AB006042.1	*NtHSP26-F*	CAGGAAGCAACAGGGCATC
AB006042.1	*NtHSP26-R*	CGGCATGTCAAAACGCATC
**Primers used for RT- PCR**		
PGSC0003DMT400002412	*StNPR1-F*	GGATTTGCTTTGCTTGCTG
PGSC0003DMT400002412	*StNPR1-R*	ACCTCGACCGGATCATTG
PGSC0003DMT40003201	*StDREB1-F*	TCGAATGCGAAGTTGGGGA
PGSC0003DMT40003201	*StDREB1-R*	AACCCATCCTTCACCGGAG
AF234297.1	*GUS-F*	CAACGTCTGGTATCAGCGC
AF234297.1	*GUS-R*	GCGGATTCACCACTTGCAA
AB158612.1	*NtActin-F*	TACGCCCTTCCTCATGCAA
AB158612.1	*NtActin-R*	ACGATCTGCAATGCCAGGA
